# The Intersection between Food Insecurity and Diabetes: A Review

**DOI:** 10.1007/s13668-014-0104-4

**Published:** 2014-10-02

**Authors:** Enza Gucciardi, Mandana Vahabi, Nicole Norris, John Paul Del Monte, Cecile Farnum

**Affiliations:** 1 School of Nutrition, Ryerson University, 350 Victoria Street, Toronto, Ontario M5B 2K3 Canada; 2 Daphne Cockwell School of Nursing, Ryerson University, 350 Victoria Street, Toronto, Ontario M5B 2K3 Canada; 3 Library and Archives, Ryerson University , 350 Victoria Street, L272-F, Toronto, Ontario M5B 2K3 Canada

**Keywords:** Coping strategies, Diabetes, Diabetes management, Diabetes prevention, Food insecurity, Food security, Hypoglycemia, Poverty, Self-care, Self-efficacy, Self-management

## Abstract

Access to sufficient, safe, and nutritious food not only affects the health of people who experience food insecurity, but also their ability to manage health conditions, such as diabetes. When people find it difficult to access sufficient food, tailoring their food selection to a diabetes regimen is even more difficult. Food insecurity in North America is consistently more prevalent among households with a person living with diabetes, and similarly, diabetes is also more prevalent in food-insecure households. Diabetes management can be stressful due to the many required responsibilities; when compounded with food insecurity, it becomes an even greater challenge. As a result, many food-insecure diabetics find themselves caught between competing priorities such as procuring food, prescribed medications and supplies for diabetes, and managing other living expenses, potentially worsening their condition and overall health. Healthcare providers should be aware and informed about the significant role that food security can play in the prevention and management of diabetes.

## Introduction

Food is a fundamental human need, and limited access to it is considered a violation of human rights [1]. Food security is an important social determinant of health [2] and ample evidence supports a direct relationship between food security and physical, mental, and social health [3–10]. The 1996 World Food Summit defined food security as everyone, at all times, having physical and economic access to sufficient, safe, nutritious food that meets their dietary needs for an active and healthy life [11]. The Summit has since added new components to this definition, such as food preferences, shifting food security away from simple access to calories and nutrition and toward access to food that is also socially and culturally acceptable. Within the context of diabetes, access to sufficient quantities of food does not necessarily guarantee food security if food quality and preferences are ignored.

Food insecurity is an international public health issue, particularly for vulnerable populations, such as female-lone-parent households and individuals who are elderly, recent immigrants, health-impaired, and/or low-income [12]. These food-insecure populations are at risk for less diverse, lower quality diets, reduced micronutrient intake, iron-deficiency anemia, and low intake of fruits and vegetables [7, 13–16]. Limited budgets among food-insecure people lead to purchasing cheaper, higher-calorie foods, which can contribute to weight gain and an increased susceptibility to one or more chronic illnesses, including type 2 diabetes [5, 6, 17–20]. Access to nutritious food not only affects people’s health, but also their ability to manage health conditions, particularly those with specific dietary regimens, [4, 6, 8, 21•] such as diabetes. When people find it difficult to access sufficient food, tailoring their food selection to diabetes regimens is even more difficult [6].

## Objective

This article synthesizes the current literature on food insecurity and diabetes self-management. This synthesis is important, because nutritional self-care is the cornerstone of diabetes management and is critical to preventing diabetes-related complications. Healthcare providers and public health policymakers need to be aware of how food insecurity and diabetes intersect, with the goal of increasing accessibility of healthy foods to prevent diabetes and better manage the condition.

## Methods

### Search Strategy

We searched Medline, CINAHL, the Cochrane Database of Systematic Reviews, Web of Science, and PsychInfo using some or all of the following keywords: *diabetes*, *diabetes mellitus, dietary intake, food access, food deserts, food insecurity, food intake, food preferences, food security, food supply, self-care, self-efficacy, self-management.* English-language articles published before May 2014 were reviewed if they (a) explored or measured food security, food augmentation strategies, food preferences, food access, and/or dietary intake; and (b) targeted a diabetic population. Of the 539 articles initially found, 16 articles met the inclusion criteria. Another 23 articles were identified by hand-searching the references of these 16 articles, for a total of 39 articles reviewed.

## Review Methods

### Problems with Data on Food Insecurity

No global consensus has been reached on how to define or measure food insecurity. Quantitative data come primarily from telephone surveys, some more standardized than others, such as the US Department of Agriculture Household Food Security Survey Module (USDA HFSSM) [22, 23]. Qualitative data from interviews help clarify personal experiences of food insecurity. Most of the articles reviewed used the USDA HFSSM or a slightly modified version of it [6, 21•, 24, 26, 27••, 28, 29•, 30, 31••, 32, 33•, 34–36], and only a few studies had developed their own questionnaires [37•, 38]. The authors of one article used the Household Food Insecurity Access Scale (HFIAS), a measure of food accessibility developed by the US Agency for International Development and validated for use in developing countries [39•]. However, scholars have identified the need to develop culturally relevant, globally appropriate measures that take into account typical local foods, as well as meal sizes and frequency [40]. Of the reviewed articles, four used qualitative methods, 26 used quantitative methods, and three used mixed methods. Regardless of the approach, all research is limited by the respondents’ understanding of key terms such as food insecurity. Even when participants are provided with a definition, their own perceptions of this term, imbued with their own experiences of food insecurity and the shame of being unable to support themselves or their families, can influence their responses. Thus, surveys may underestimate food insecurity among vulnerable populations. Similarly, large national surveys based on self-reporting may underestimate the prevalence of diabetes in food-insecure households [35]. National surveys often exclude or underrepresent populations at greater risk for food insecurity or diabetes (i.e., Aboriginal peoples on reserves, homeless persons) [6, 24, 32]. Together, these factors may underestimate diabetes and food insecurity and limit examinations of how these interact.

## Findings

### Prevalence of Food Insecurity and Diabetes

Household food insecurity in North America is consistently more prevalent among households with a person living with diabetes [32, 33•]. The prevalence of household food insecurity in Canada in 2005 was 9.3 % among individuals with diabetes, compared with 6.8 % among those without diabetes. Additionally, each year, the earlier a person is diagnosed with diabetes the likelihood of household food insecurity increases by 4 % [32]. Similarly, diabetes is more prevalent in food-insecure households [24, 35]. From 1999–2004, the prevalence of diabetes in the US was 10.2 % in food-insecure households, compared with 7.4 % in food-secure households [35]. Not only do the prevalence rates of diabetes and food insecurity mirror each other, but diabetes prevalence also rises with increasing severity of food insecurity (10 % for mild household food insecurity vs. 16.1 % for severe) [24, 35].

Gender and age differences appear at the intersection of diabetes and food insecurity. More women than men with diabetes in Canada are food-insecure (12 % vs. 7 %, respectively) [32]. In Nova Scotia, household food insecurity prevalence is dramatically higher in households with children diagnosed with diabetes (21.9 %), compared with household food insecurity prevalence among adults in Nova Scotia and Canada-wide (14.6 % and 9.2 %, respectively)[31••]. Additionally, homebound elders with diabetes are twice as likely to be food-insecure than elders without diabetes [38]. As expected, household food insecurity is much more prevalent in more impoverished areas (e.g., rural Appalachia in the US) and the joint prevalence of household food insecurity and diabetes is also more prevalent in these areas compared with food-secure households (38 % vs. 26 % )[34]. Finally, food insecurity among diabetic populations in developing countries is of increasing concern as the prevalence of both diabetes and hunger continues to rise in these regions [39•, 41, 42].

### Intersection of Food Security and Diabetes

Recent research suggests that food insecurity is a risk factor for developing diabetes [24, 43]. A large-scale longitudinal survey conducted in the US revealed that diabetes risk was approximately 50 % higher among adults in food-insecure households than in food-secure households [35]. Food-insecure adults are two to three times more likely to have diabetes than adults who are food-secure, even after controlling for important risk factors such as income, employment status, physical measures, and lifestyle factors [21•, 24]. Furthermore, more food-insecure pregnant women are at risk for gestational diabetes than those who are food-secure [44]. Diets common in food-insecure households (e.g., inexpensive carbohydrates) may increase dietary glycemic load, and therefore, increase the risk of diabetes [35]. However, more longitudinal studies are needed to confirm food insecurity as a risk factor for diabetes.

### Effects of Food Insecurity and Diabetes

#### Food Purchase, Planning, and Preparation

Diabetes management can be stressful due to its many required responsibilities, and when compounded with food insecurity, it becomes an even greater challenge. Coping strategies reported by food-insure persons living with diabetes include skipping meals, cutting meal size, and eating stale food [31••, 33•, 45, 46]. Some people purchase cheaper or canned food, buy in bulk, buy certain items only on sale, eat seasonal foods, freeze foods, comparison shop, group shop with neighbors, or receive food from relatives. Adults may eat less food so that their children have enough [31••] and may use community-based food programs (e.g., meal assistance programs) [46]. Many of these strategies require planning and practical skills to manage under difficult circumstances [46, 47].

People living with food insecurity often have limited control over their living environments, which can affect their ability to access and prepare healthy food. Often the only stores in low-income neighborhoods are convenience stores, which carry low-nutrition, high-cost foods [35, 48]. It can also be difficult to find stable accommodations with access to a stove and a refrigerator, which are needed to prepare meals at regular intervals to manage diabetes [25, 49]. As a result, individuals often choose easier-to-access food (e.g., canned, pre-cooked), which tends to be energy-dense, high in salt, and detrimental to those living with diabetes [25]. Diabetes management is difficult for individuals relying on free food available at food banks, shelters, or drop-in meals: this food is usually not suitable for a diabetes diet [47, 49, 50], and is often canned and high in salt, starch, and sugar [47, 50].

#### Diabetes Management

Food insecurity interferes with following diabetes self-management recommendations [51]. Researchers consistently report an association between food insecurity and low self-efficacy to manage diabetes [25, 27••, 28, 51]. The extra out-of-pocket healthcare expenses [52], such as purchasing prescribed medications and supplies [50] exacerbate food insecurity (31). Food-insecure people are more likely to delay filling prescriptions [53], re-use needles, and/or monitor their blood glucose less often due to the cost of supplies (i.e., test strips, meters, batteries) (31, 29•, 49). Many must choose between buying healthy foods, diabetes medications and supplies, and paying rent [28, 35, 50]. Many forego medication to afford food [54, 55], but more than one-third of diabetics who visit food banks or soup kitchens pay for medication before food [56]. Ultimately, the choice between food and medication predisposes individuals either to hypoglycemia (if medications are taken instead of food) or hyperglycemia (if food is eaten instead of medications taken) [10, 28, 54]. The literature confirms the clinical effects of these decisions: the following subsections describe the cumulative effects of food insecurity on diabetes management.

#### Diet

A healthy diet – high in protein, vegetables, and fruit, and low in fat and carbohydrates – is important for diabetes management. However, for food-insecure diabetics, maintaining a healthy diet is very challenging. As noted, they generally consume fewer fruits, vegetables, and protein, and rely more on energy-dense foods than food-secure diabetics [32, 50]. Sustained periods of high blood sugars can significantly hamper an individual’s capacity to manage blood sugar levels and prevent consequent complications (e.g., blindness, amputations, kidney failure).

#### Glycemic Control (A1c)

Food-insecure diabetics have marginally higher A1c levels than food-secure diabetics [27••, 28, 29•, 34–36] and are at increased risk for poorer glycemic control (A1c levels higher than 7 %), even after controlling for socio-demographic and diabetes-related factors [29•, 34, 36, 45, 51]. A gender analysis of a rural Appalachian sample in the US revealed significantly higher A1c levels among food-insecure women with diabetes than among food-secure women [34]. A study conducted in Nova Scotia revealed a similar pattern among children with diabetes: mean A1c levels were higher in food-insecure households than in food-secure households (9.5 % vs. 8.96 %) [31••].

#### Hypoglycemia

Food-insecure diabetics are at risk of clinically significant hypoglycemia, a complication of diabetes treatment that may affect long‐term cognitive function and can occasionally be fatal [57]. Seligman et al. observed twice as many hypoglycemic episodes [29•] and more hypoglycemia-related emergency department visits among food-insecure diabetics than among food-secure diabetics [28]. Similarly, clinical studies have reported elevated risk for hypoglycemia among low-income diabetics [28, 29•]. Among food-insecure diabetics, far more hypoglycemic episodes are attributed to the inability to afford food, than among food-secure diabetics (43.2 % vs. 6.8 %) [29•]. Diabetics who take insulin or oral medications to lower blood sugars often report more hypoglycemia [58] during times of reduced food intake [59].

#### Weight Status

Reports of the association between weight status and food insecurity in the diabetes population are inconsistent and may vary between developed and developing countries. A study conducted in Jordan reported that obese individuals with diabetes who were severely food-insecure had a significantly higher average body mass index (BMI), even though they consumed fewer calories than mildly food-insecure or food-secure individuals [45]. Similarly, among rural older adults with diabetes in the US, participants who reported mild food insecurity had on average a higher BMI than those who were food secure (35.6 vs. 30.6) [46]. However, among diabetes patients studied in multiple clinics in western Kenya, the food-insecure group had a lower average BMI compared with the food-secure group [39•]. Other studies found no differences in BMI among adults [32] or children [31••] with diabetes in food-secure and food-insecure households.

#### Preventing Comorbities

Preventing diabetes‐related complications requires controlling not just blood sugar, but also blood pressure and cholesterol primarily to prevent cardiovascular disease [60]. Researchers have inconsistently reported that individuals living in food-insecure households are at increased risk for high blood pressure, cholesterol, and heart disease [6, 34, 35]. However, little research has focused on the possibility of increased risk among food-insecure individuals with diabetes. In a representative sample of Americans with diabetes, food-insecure adults were more likely to have poor low-density lipoprotein cholesterol control compared to food-secure adults (68.8 % vs. 49.8 %), while blood pressure control appeared to be unaffected by food security, even after adjusting for socio-demographic and clinical factors [36]. Among food-insecure adults, difficulties paying for medications may contribute to poor control of blood pressure, cholesterol, and depression, each of which may increase diabetes complications [57].

Tobacco use and physical inactivity are critical risk factors for cardiovascular complications of diabetes. Food-insecure individuals with diabetes reportedly smoke more than food-secure individuals [21•, 32, 35]. Food-insecure adults are also less likely to engage in physical activity than adults in food-secure households with diabetes [32]. One reason for this may be the fatigue associated with food shortages, which can make physical activity challenging [10].

#### Healthcare Usage

Studies conducted in North America have revealed no differences between food-insecure and food-secure individuals in the receipt of diabetes-specific care, such as annual A1c tests, dilated eye exams [32, 53], urine tests, or foot exams [32, 53], even after adjusting for socio-demographic factors and health status. However, after controlling for socio-demographic factors, social resources, and comorbidities, but not disease severity, food-insecure individuals with diabetes report more physician encounters in person or by phone than those who are food-secure [61]. Food-insecure people with diabetes are also more likely to report more overnight hospitalizations and emergency-room visits given their increased risk for hypoglycemia [28, 29•]. Another study of households with children with type-1 or type-2 diabetes requiring insulin found no difference in the number of emergency-room visits between food-insecure and food secure households; however, it reported that children in food-insecure households had been hospitalized more than four times as often as children in food-secure households during the previous year [31••]. Adults and children with diabetes living in food-insecure households report more unmet healthcare needs than those living in food-secure households in Canada [31••, 32].

#### Mental Health

Food insecurity does not only affect physical health; it affects overall wellbeing. For example, research findings are very consistent regarding the detrimental effects of food insecurity on mental health. Food-insecure adults with diabetes are more likely than food-secure adults with diabetes to report fair or poor overall health [32, 61], mental health, satisfaction with life, and self-perceived stress [32]. Similarly, food-insecure individuals with diabetes report more emotional distress related to diabetes than food-secure individuals with diabetes [51]. Constrained budgets, diabetes symptoms and/or complications, and the perceived interference of diabetes with daily life all contribute to higher rates of depression among food-insecure diabetics [62]. Furthermore, food insecurity is often accompanied by anxiety, worry, shame, guilt, feelings of powerlessness, and intensifying depressive symptoms [49, 63]. Food-insecure individuals with diabetes report more mood disorders [32] and depressive symptoms [21•] than food-secure individuals with diabetes. Social support reportedly buffers the negative emotions associated with food insecurity among individuals with diabetes [62]. This buffering effect may be related to family and friends alleviating major stressors by providing resources that protect mental health, such as food, money, or emotional support [50].

### Recommendations for Healthcare Providers

#### Screening for Food Insecurity

Healthcare providers should be aware of the significant role that food insecurity can play in the identification of diabetes and early interventions to prevent and manage the disease [33•, 37•, 38, 64]. Some authors have suggested that household food insecurity and its severity should be included in diabetes patient assessment and diabetes care plans, while others [29•, 33•] have advocated food-insecurity screening for all patients [37•]. Routine food-insecurity screening may help healthcare providers devise realistic and personalized management interventions, such as cost-neutral food strategies, medication regimes that reduce the risk of hypoglycemic episodes [28], referring patients to local or national food programs, and providing free diabetic supplies to mitigate food insecurity [28, 33•, 36, 37•, 48, 65, 66].

Screening for food insecurity should be non-judgmental, especially in the context of a patient–provider relationship, to reduce the risk of patients feeling embarrassed or ashamed, and thereby encouraging them to be truthful [37•]. To ascertain whether a patient is likely to be at risk of food insecurity, healthcare professionals can ask patients to respond to a single question: “In the past month, was there any day when you or anyone in your family went hungry because you did not have enough money for food?” Use of this question appears to yield acceptable validity for use in clinical settings [67].

#### Nutritional Counselling

When making recommendations about dietary change, healthcare providers should consider each patient’s resources for purchasing, preparing, and cooking food; nutritional counselling for food-insecure individuals with diabetes should be tailored to fit their incomes and living circumstances. Providers should explore challenges that may hinder a patient’s ability to follow the prescribed therapeutic diet [24, 53, 64]. Patients only use the food options that are available to them; these are not only shaped by economic circumstances, but also by ethno-cultural identities [26]. For certain ethno-cultural groups, it may be necessary to develop an individualized nutrition plan that suits the whole family [26]. Furthermore, dietary advice should include a discussion of how to adhere optimally to therapeutic diets when clients cannot afford their preferred foods and how to best control blood sugars when access to food is limited [33•].

Healthcare providers should urge patients to shift their dietary intake away from inexpensive carbohydrates and fats and toward vegetables, fruits, protein, and dairy products, while acknowledging limited budgets [33•, 43]. Encouraging the intake of frozen fruits and vegetables (rather than canned) is an inexpensive way to reduce significantly the intake of salt and sugar, which are commonly added to canned foods [57]. Dietary counseling should also emphasize cost-neutral strategies, such as smaller portion sizes, rather than food substitutions [28]. Discussing portion sizes of food items that are culturally preferable and acceptable to patients and their families may be as important as recommending foods that are affordable [26]. Providers can also help patients identify resources in their neighborhoods and help them find stores selling healthier foods at lower prices [37•]. Finally, print resources intended for food-insecure individuals with diabetes should acknowledge that healthy eating is not merely about finding low-cost food, and include strategies for coping with limited food budgets while adhering to therapeutic diet as best as possible [33•].

## Medications and Reducing Hypoglycemic Episodes

Healthcare providers can suggest that patients treat days when they have poor access to food as a ‘sick or fasting day,’ and take less medication that could cause hypoglycemia if their food intake will be low [37•]. Additionally, treatment regimens should include medications that involve less risk of hypoglycemia when food access is unpredictable [57]. For example, Metformin and DPP-4 inhibitors involve a very low risk of hypoglycemia when used alone [68]. For patients requiring insulin, regimens that allow for as much flexibility (such as multiple daily injections with basal and bolus insulin) can allow patients to omit doses if meals are missed, reducing the risk of hypoglycemia [69]. Glycemic targets may also need to be adjusted upward to mitigate the elevated risk of hypoglycemia associated with food insecurity [28]. When prescribing oral antihyperglycemic agents or insulin, healthcare providers should consider drug formulary coverage, the type of drug coverage the patient has, and the cost of medications, with an emphasis on patient safety and reduced hypoglycemia risk [57]. In some cases, certain medications can be obtained through compassionate or assistance programs [57].

## Diabetes Self-management Education

Diabetes self-management support can effectively improve self-care behaviors and glycemic control among food-insecure individuals. One study found that after self-management education targeting food-insecure and food-secure individuals with diabetes, the A1C and self-efficacy levels of the food-insecure individuals improved significantly more over time than those of food-secure individuals [27••]. These findings suggest that food-insecure participants benefitted from diabetes education that focused on self-management strategies (including dietary changes), even though the intervention did not specifically address low-budget strategies. Lacking nutrition knowledge has been associated with an increased likelihood of mild, but not severe, household food insecurity [21•]. This result suggests that, although some nutritional knowledge may protect against milder forms of food insecurity, barriers faced by people with more severe food insecurity cannot be addressed with nutrition knowledge alone. Self-management education programs should include a component that focuses on strategies for coping with food insecurity.

## Policy Recommendations

Poverty is at the root of food insecurity, but policies to address poverty are challenging to develop and implement because it intersects with other social determinants of health, such as employment, education, and housing. Nonetheless, it is important to recognize the challenges that low-income individuals and families face in managing chronic conditions. In addition to focusing on diabetes prevention and management [24], policymakers need to focus on reducing food insecurity. Social policies aimed at expanding access to employment, housing, and food for people living in poverty may improve the ability of low-income people with diabetes to manage their disease and maintain their overall health [25].

Increased access to food and use of food and nutrition assistance programs can reduce food insecurity. Programs can reduce the financial burden of healthy food by offering vouchers for fresh fruits and vegetables, subsidizing fresh fruits and vegetables so they are cheaper than high energy-density carbohydrate-laden food, and delivering affordable fresh fruits and vegetables to low-income areas, particularly to home-bound recipients [35]. Such programs should collaborate with neighborhood community organizations and food retailers to improve the availability of healthy food; for example, by providing financial incentives to stores in low-income neighborhoods to stock healthy low-cost foods or to grocery chains to open stores in ‘food deserts’. Additionally, fruit and vegetable trucks, stands, or farmers markets could help distribute fresh produce more easily, and community gardens can allow residents to grow their own vegetables and fruit [66].

Policies that improve access to affordable or subsidized diabetic supplies for those who cannot afford them can also minimize the effects of food insecurity on diabetes self-management [32]. Ultimately, however, innovative programs to promote and support economic self-sufficiency for individuals, families, and households are the best way to reduce food insecurity [34, 70].

## Recommendations for Future Research

This literature review identified various avenues for future research to better understand and mitigate the challenges faced by food-insecure individuals with diabetes. First, more research is needed to clarify how these individuals make challenging decisions and compromises regarding food purchases and medical supplies, particularly during periods of monthly income scarcity [30]. Second, more longitudinal, prospective, population-based studies with large samples are needed to clarify how food insecurity affects diabetic individuals, particularly with regard to glycemic control and long-term complications and health outcomes [31••]. Third, researchers need to focus on effective strategies for counselling low-income diabetes patients to help them achieve healthier diets [24, 51]. Fourth, healthcare providers should investigate their role in monitoring and addressing household food insecurity among clients for whom better nutrition is a key component of disease management. Fifth, more systematic ways are needed to measure food insecurity among individuals accessing the healthcare system for treatment of chronic diseases, and thereby evaluate how best to meet their needs and improve their health outcomes [33•].

## Conclusions

For those living with diabetes, food insecurity can add to the negative effects of diminished health and social wellbeing. The available literature strongly suggests that multi-factorial approaches at both the community and system levels are needed to mitigate the effects of food insecurity, including its effects on diabetes management. Screening for food insecurity in healthcare settings is one important step toward devising realistic, personalized diabetes management interventions. Policies targeted at improving the financial power of people with diabetes to access sufficient, safe, nutritious, and culturally acceptable foods are imperative.
